# Phase I study of miriplatin combined with transarterial chemotherapy using CDDP powder in patients with hepatocellular carcinoma

**DOI:** 10.1186/1471-230X-12-127

**Published:** 2012-09-20

**Authors:** Kenya Kamimura, Takeshi Suda, Yasushi Tamura, Masaaki Takamura, Takeshi Yokoo, Masato Igarashi, Hirokazu Kawai, Satoshi Yamagiwa, Minoru Nomoto, Yutaka Aoyagi

**Affiliations:** 1 Division of Gastroenterology and Hepatology, Graduate School of Medical and Dental Sciences, Niigata University, Niigata, Japan

**Keywords:** Miriplatin, Hepatocellular carcinoma, Cisplatin powder, Phase I clinical trial

## Abstract

**Background:**

There is no standard therapeutic procedure for the hepatocellular carcinoma (HCC) in patients with poor hepatic reserve function. With the approval of newly developed chemotherapeutic agent of miriplatin, we have firstly conducted the phase I study of CDDP powder (DDP-H) and miriplatin combination therapy and reported its safety and efficacy for treating unresectable HCC in such cases. To determine the maximum tolerated dose (MTD) and dose-limiting toxicity (DLT) for the combination of transarterial oily chemoembolization (TOCE) and transarterial chemotherapy (TAC) using miriplatin and DDP-H for treating unresectable hepatocellular carcinoma (HCC).

**Methods:**

Transarterial chemotherapy using DDP-H was performed through the proper hepatic artery targeting the HCC nodules by increasing the dose of DDP-H (35–65 mg/m^2^) followed by targeting the HCC nodules by transarterial oily chemoembolization with miriplatin.

**Results:**

A total of nine patients were enrolled in this study and no DLT was observed with any dose of DDP-H in all cases in whom 80 mg (median, 18–120) miriplatin was administered. An anti-tumour efficacy rating for partial response was obtained in one patient, while a total of four patients (among eight evaluated) showed stable disease response, leading to 62.5% of disease control rate. The pharmacokinetic results showed no further increase in plasma platinum concentration following miriplatin administration.

**Conclusion:**

Our results suggest that a combination of DDP-H and miriplatin can be safely administered up to their respective MTD for treating HCC.

**Trial registration:**

This study was registered with the University Hospital Medical Information Network Clinical Trials Registry (UMIN-CTR000003541).

## Background

Hepatocellular carcinoma (HCC) is the most common type of liver cancer [1] and various therapeutic options have been developed by focusing on the specific tumour stage and hepatic functional reserve [2-9]. A variety of transarterial treatments have been provided to cases at relatively advanced stages [3], and these treatments were roughly divided into the following three groups: tran-sarterial chemoembolization (TACE), transarterial oily chemoembolization (TOCE) and transarterial chemotherapy (TAC), based on the likelihood of deteriorating hepatic reserve. TACE involves hepatic arterial injections of chemotherapeutic agents combined with embolizing materials. TOCE is solely an arterial administration of a combination of chemotherapeutic agents and oily contrast medium of lipiodol ultra fluid (Laboratory Guerbet, Aulnay-sous-Bois, France), while in TAC, chemotherapeutic agents alone are infused through the hepatic artery. Although TACE is only a transarterial procedure, for which therapeutic efficacy has been proved in randomised prospective controlled studies, the deterioration of hepatic reserve is estimated at 20%–58%, mainly because of ischaemic damage to the nontumourous background liver [10,11], inferring a higher risk of unfavourable reduction in hepatic reserve function in cases with poor hepatic reserve. Therefore, to develop a safe and efficient transarterial therapeutic procedure in such cases, other effective means of performing TOCE, TAC, and TOCE + TAC have been tested [5,12-15].

TACE and TOCE were recently compared in a randomised phase III trial using zinostatin stimalamer dissolved in lipiodol [12] with subsequent arterial embolization (TACE) or without embolization (TOCE). Interestingly, the results showed no improvement in survival rates by performing embolization and TOCE represented to be a therapeutic option for HCC patients with low hepatic reserve. However, two major concerns with TOCE are: 1) the method of combining water-based chemotherapeutic agents with oily lipiodol in a stable formulation; and 2) that TOCE is unable to target wide area of the liver as it reduces the hepatic arterial flow, although tentative, that may result in hepatic failure. For first concern, Miriplatin, a third-generation platinum derivative with lipophilic moiety that forms a suspension with lipiodol, was recently developed and approved for clinical use in Japan as a novel chemotherapeutic agent for HCC [16-21] with promising results [22-24]. For second concern, as TAC requires no embolization, that can be injected in wide area and its anti-tumour effect has been reported in several studies [5,13-15], followed by the promising results from a multicentre phase II study in patients with unresectable HCC using cisplatin (CDDP), a first-generation platinum agent, in which the response rate was recorded as 33.8% [13], it might be effective to treat wide area of the liver with poor hepatic reserve function. In addition, the first-pass kinetics [25] of CDDP by TAC contribute to the anti-tumor effect and decrease the adverse systemic events [5]. Since highly concentrated CDDP powder for TAC (DDP-H, IA-call^®^; Nippon Kayaku Co., Ltd) is available in Japan, TAC is now widely used in Japan to treat multiple small tumours or patients with poor hepatic reserve [5,13,26].

Based on these results and the advances in the development of new chemotherapeutics, it is reasonable to consider the combination therapy of CDDP-TAC with miriplatin-TOCE to treat advanced stage HCC with poor hepatic reserve function safely and effectively. Therefore, in this study we conducted a phase I dose-escalation study on DDP-H-TAC followed by miriplatin-TOCE to determine the maximum tolerated dose (MTD) and dose-limiting toxicity (DLT) in unresectable HCC. The safety issue with regard to the combination of two platinum-based chemotherapeutic agents will be discussed by referencing the pharmacokinetics of platinum.

## Methods

### Patient selection

Patients with HCC were considered eligible for the study if they fulfilled the following criteria: 20–80 years of age; at least one measurable tumour blush on angiography; histologically and/or clinically diagnosed HCC; no other therapeutic treatment was found to be effective or appropriate to their condition, according to the Japanese guidelines for HCC treatment; an Eastern Cooperative Oncology performance status of 0–2; adequate hepatic function (Child–Pugh, score ≤7; total bilirubin, ≤3.0 mg/dl; albumin, ≥3.0 g/dl); adequate haematological function (neutrophils, ≥1,500/mm^3^; platelets, ≥50,000/mm^3^; haemoglobin, ≥8.0 g/dl); adequate renal function (creatinine clearance, ≥50 ml/min adjusted for 1.73 m^2^ of body surface area); serum amylase, ≤324 IU/dl and an interval of 4 weeks or more since previous therapy.

All nodules were radiologically diagnosed as HCC when they satisfied at least one of the following criteria based on CT or MRI: typical haemodynamics of classical HCC (substantial enhancement during arterial phase followed by a washout with ‘corona-like’ peripheral enhancement in equilibrium phase) and similar characteristics of coexisting nodules that had been diagnosed as HCC. All eligible HCC cases were recurrent with a history of CDDP administration in eight patients. Patients with the following characteristics were considered ineligible: massive pleural effusion and/or ascites refractory to treatment; active cancer other than HCC; active infectious disease; active haemorrhagic state; severe mental disorder; hepatic encephalopathy; history of allergic reaction to iodine phase contrast and/or platinum agents; ongoing interferon therapy and difficulty with oral food intake. This study was approved by the institutional review board of Niigata University Hospital and was registered with the University Hospital Medical Information Network Clinical Trials Registry (UMIN-CTR 000003541). Written informed consent was obtained from all patients and the study protocol conformed to the ethical guidance of the 1975 Declaration of Helsinki.

### Method of administration

CDDP powder, DDP-H (Nippon Kayaku Co., Ltd. Tokyo, Japan), was solubilised in saline at a concentration of 100 mg/70 ml immediately before use and infused into the entire liver through the proper hepatic artery at a rate of 126 ml/h, providing in total 35 mg/m^2^. This was followed by TOCE with miriplatin, prepared according to the instructions, through the nutrient vessels of the target tumour using a maximal dose showing corresponding drainage portal veins up to a volume of 6 ml. If no DLT was recorded, the same regimen was carried out by increasing DDP-H by 15 mg/m^2^, based on the modified Fibonacci method in which DLT is defined as adverse events of grade ≥3 in nonhaematological or grade ≥4 in haematological toxicity, according to the NCI-CTCAE version 4.0. If any of the three patients showed as having DLT, three more patients were enrolled. MTD was judged to have been exceeded when two patients showed DLT. MTD was defined as the maximum dose where no more than two of the six patients experienced DLT. If two or more cases were already suffering from DLT at the initial dose of 35 mg/m^2^, this dose was reduced by 10 mg/m^2^ to 15 mg/m^2^.

### Evaluation of anti-tumour effects

Anti-tumour response was evaluated from CT images obtained before and 3 months after treatment. Evaluation was performed in accordance with the modified Response Evaluation Criteria in Solid Tumors (RECIST) guideline, a new response evaluation criteria in solid tumours [27]. The tumour markers of AFP and DCP were followed at appropriate time periods for each patient.

### Platinum pharmacokinetics

Total plasma platinum concentration was measured and pharmacokinetic evaluation performed for all patients. Plasma samples were collected in heparinised tubes at 24 h and 7 days following the administration of DDP-H and miriplatin. As reference, 50 mg/m^2^ (80 mg/body) of CDDP in liquid form was administered through the proper hepatic artery for the entire liver at a rate of 1 mg/min, and the concentration was quantified before the administration and at 0.5, 1.0, 1.5, 2, 4, 12 and 24 h after administration. Plasma platinum concentration was measured by atomic absorption spectrometry (Nac Co., Ltd., Tokyo, Japan).

## Results

### Patient characteristics

A total of nine eligible patients were enrolled in this study from July to October 2010 and divided into three groups; none of the three patients from each group developed DLT at DDP-H dose levels of 35 (level 1), 50 (level 2) and 65 (level 3) mg/m^2^. Patient characteristics before treatment are summarised in Table 1. Performance status was 0 in eight patients and 1 in one patient (case 1). The aetiology of liver cirrhosis was HBV infection (*n =* 1), HCV infection (*n* = 4), alcoholic abuse (*n* = 3) and autoimmune hepatitis (*n* = 1). Residual liver function was relatively good with a median Child–Pugh score of 6, eight patients in grade A and one in grade B, and no marked renal dysfunction was observed. All patients had a history of HCC treatment; eight patients, other than case 3, had a history of DDP-H-TAC followed by epirubicin-TOCE.

**Table 1:** Patient characteristics

**Group**	**Level 1**			**Level 2**			**Level 3**		
**CDDP (mg/m**^**2**^**)**	**35**			**50**			**65**		
**Case number**	**1**	**2**	**3**	**4**	**5**	**6**	**7**	**8**	**9**
**Age (years)**	80	62	80	78	61	80	63	79	80
**Gender (M, Male/F, Female)**	M	M	M	M	M	M	M	F	F
**Performance status**	1	0	0	0	0	0	0	0	0
**HBV infection**	-	-	-	-	-	-	+	-	-
**HCV infection**	+	+	-	-	-	+	-	+	-
**Alcohol**	-	-	+	+	+	-	-	-	-
**Autoimmune hepatitis**	-	-	-	-	-	-	-	-	+
**Child-Pugh Score**	6	6	5	6	6	7	5	6	6
**Recurrence (Y, Yes/N, No)**	Y	Y	Y	Y	Y	Y	Y	Y	Y
**Interval to previous therapy (M)**	6	8	6	23	10	21	13	19	3
**Previous therapy**	TACE	TAC	TACE	TACE	TAC	TACE	TAC	TACE	TACE
**History of CDDP Administration**	Y	Y	N	Y	Y	Y	Y	Y	Y
**Number of tumors**	3	2	1	>5	>5	4	4	>5	>5
**Maximum tumor size (mm)**	15	15	14	20	10	34	24	10	30
**Vascular invasion (Y, Yes/N, No)**	N	N	N	N	N	N	N	N	N
**Metastasis (Y, Yes/N, No)**	N	N	N	N	N	N	N	N	N
**Stage (UICC)**	II	II	I	II	II	II	II	II	II
**Tumor location (PAMLC)**	PA	ML	ML	AM	ML	M	P	A	PA
**BSA (m**^**2**^**)**	1.486	1.6	1.457	1.5	1.72	1.68	1.415	1.538	1.538
**Ccr (ml/min)**	68	118	75	89	121	92	83	95	85
**CDDP (mg/body)**	52	56	51	75	86	84	92	100	100
**Miriplatin (mg/body)**	86	18	80	120	60	60	74	100	120

TACE, transarterial chemoembolization; TAC, transarterial chemotherapy. Tumour location: P, posterior segment; A, anterior segment; M, medial segment; L, lateral segment; C, caudal segment. BSA, body surface area; Ccr, creatinine clearance.

The total dose of DDP-H administered was 51, 52 and 56 mg/body at level 1; 75, 84 and 86 mg/body at level 2 and 92, 100 and 100 mg/body at level 3. Total dose of miriplatin administered was 18, 80 and 86 mg at level 1; 60, 60 and 120 mg at level 2 and 74, 100 and 120 mg at level 3. All nine patients were assessed for toxicity of CDDP combined with miriplatin and for the pharmacokinetics of plasma platinum concentration. One patient underwent radio frequency ablation (RFA) before response evaluation, and thus eight patients were assessed for anti-tumour response.

### Toxicity

Haematological and nonhaematological toxicity in all patients was evaluated using NCI-CTCAE (National Cancer Institute Common Terminology Criteria for Adverse Events) version 4.0, summarised in Table 2. No grade ≥3 in nonhaematological or grade ≥4 in haematological toxicity was observed. One patient (case 4 in the level 2 group) developed grade 3 neutropenia (reduced from 3000/mm^2^ to 1710/mm^2^, 6 weeks after injection) and subsequently recovered over 2 weeks. All three groups showed a grade 2 increase in aspartate aminotransferase and alanine aminotransferase (cases 3, 5, 6 and 9) and grade 1–2 hypoalbuminaemia (cases 1, 2, 5, 7 and 9). No marked increase was noted in creatinine, except in case 7, which showed a transient increase of 1.13 times higher than baseline level 4 days after the administration of 65 mg/m^2^ CDDP combined with 74 mg/body miriplatin. The most frequent adverse event was grade 1 monophasic fever, which was observed in cases 1, 4, 8 and 9 receiving 86, 120, 100 and 120 mg/body of miriplatin, respectively. Therefore, in this clinical study, the MTD of CDDP in combination with miriplatin was determined as 65 mg/m^2^, which is the maximum dose for DDP-H-TAC monotherapy.

**Table 2:** Haematological and nonhaematological toxicity

	**Level 1**				**Level 2**				**Level 3**			
	**n = 3**				**n = 3**				**n = 3**			
	**Grade**											
**Hematological toxicity (grade)**	**1**	**2**	**3**	**4**	**1**	**2**	**3**	**4**	**1**	**2**	**3**	**4**
**White blood cell decreased**	0	1	0	0	0	0	1	0	0	0	0	0
**Neutrophil count decreased**	0	1	0	0	0	0	1	0	0	0	0	0
**Platelet count decreased**	0	1	0	0	0	0	0	0	2	0	0	0
**Anemia**	0	1	0	0	0	0	0	0	0	0	0	0
**Nonhematological toxicity (grade)**	**1**	**2**	**3**	**4**	**1**	**2**	**3**	**4**	**1**	**2**	**3**	**4**
**AST increased**	0	1	0	0	1	1	0	0	0	1	0	0
**ALT increased**	0	1	0	0	1	1	0	0	0	1	0	0
**Blood bilirubin increased**	0	0	0	0	1	0	0	0	1	0	0	0
**INR increased**	0	0	0	0	0	0	0	0	0	0	0	0
**Hypoalbuminemia**	0	2	0	0	0	1	0	0	1	0	0	0
**Creatinine increased**	0	0	0	0	0	0	0	0	1	0	0	0
**Anorexia**	0	0	0	0	0	0	0	0	1	0	0	0
**Nausea**	0	0	0	0	0	0	0	0	0	0	0	0
**Vomiting**	0	0	0	0	0	0	0	0	0	0	0	0
**Fever**	1	0	0	0	1	0	0	0	2	0	0	0
**Diarrhea**	0	0	0	0	0	0	0	0	0	0	0	0
**Fatigue**	0	0	0	0	0	0	0	0	1	0	0	0
**Alopecia**	0	0	0	0	0	0	0	0	0	0	0	0
**Urticaria**	0	0	0	0	0	0	0	0	0	0	0	0
**Abdominal pain**	0	0	0	0	0	0	0	0	0	0	0	0

National Cancer Institute Common Terminology Criteria for Adverse Events version 4.0 was applied to evaluate toxicity.

### Pharmacokinetics of platinum

**Figure 1:**
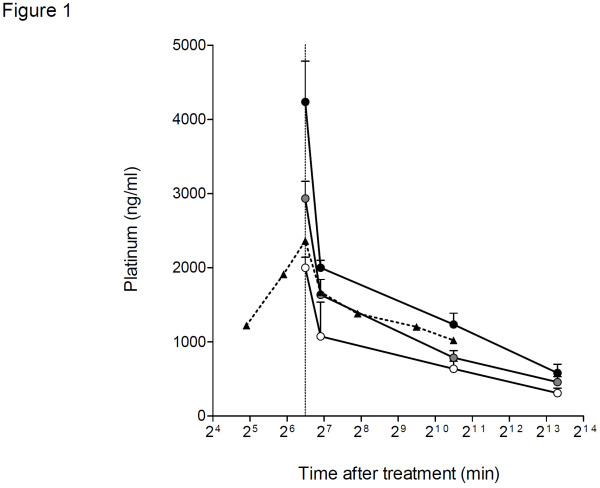
**Platinum pharmacokinetics.** Platinum concentration was measured in all patients at three levels. Level 1, white circle; level 2, grey circle and level 3, black circle. Plasma platinum concentration was also measured in a 63-year-old male patient during and after administration of CDDP (50 mg/m^2^ or 80 mg/body weight) for HCCs through the proper hepatic artery at a concentration of 0.5 mg/ml and at a flow rate of 1 mg/min (black triangle with broken line)

To examine whether additional miriplatin following DDP-H administration further increases plasma platinum concentration, plasma samples were collected for pharmacokinetic studies from all nine patients at appropriate time periods after the administration of these agents. Since total platinum concentration in peripheral plasma during and after TAC in a control case, using 50 mg/m^2^ of CDDP administered through the hepatic artery, peaked at the end of TAC and gradually decreased over the following 2 days (Figure 1), the plasma platinum concentration was evaluated at the end of DDP-H-TAC and miriplatin-TOCE and 24 h and 7 days after the initiation of DDP-H-TAC. At the end of DDP-H-TAC, median Cmax for level 1, 2 and 3 groups was 2000, 2933 and 4233 ng/ml, respectively. No further increase was detected following the administration of miriplatin: the plasma platinum concentration gradually decreased over 7 days to 310, 456 and 580 ng/ml in level 1, 2 and 3 groups, respectively. These results indicate that the concentration of platinum in the plasma showed no substantial increase with the addition of miriplatin to CDDP administration, as expected.

### Anti-tumour effects

**Figure 2:**
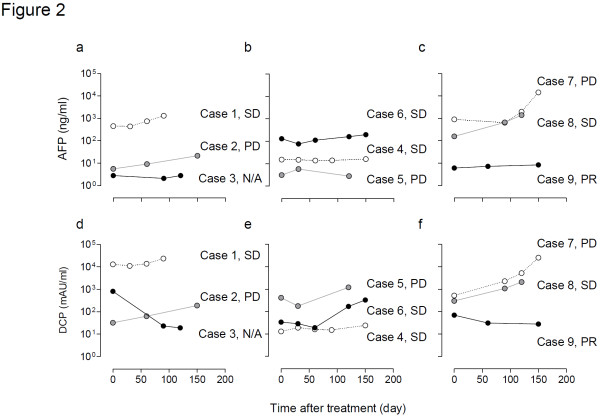
**Anti-tumour effects: levels of tumour markers.** Time-dependent levels of α-fetoprotein (**a**–**c**) and des-γ-carboxy prothrombin (**d**–**f**) after combination therapy of IA-call^®^ and miriplatin at levels 1 (**a**, **d**), 2 (**b**, **e**) and 3 (**c**, **f**). Tumour markers are represented as white circles in cases 1, 4 and 7; grey circles in cases 2, 5 and 8 and black circles in cases 3, 6 and 9. PR, partial response; SD, stable disease; PD, progressive disease; N/A, not applicable for the response evaluation

**Figure 3:**
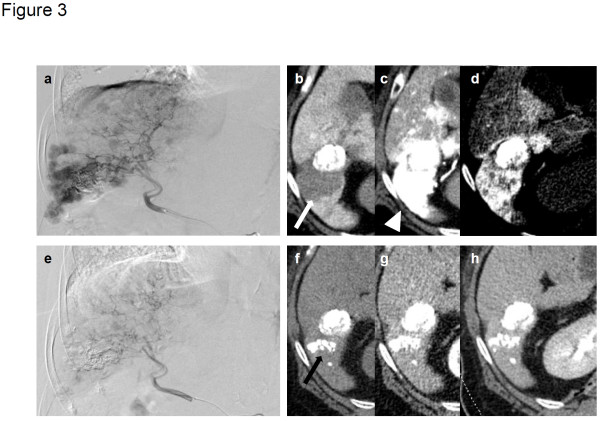
**Representative images of tumour from case 9 showing partial response after administration of DDP-H TAC and miriplatin-TOCE.** Before treatment: **a**, right hepatic angiography; **b**, computed tomography during arterial portography (CTAP). White arrow indicates tumour defect on CTAP; **c**, early phase of CT hepatic arteriography (CTHA); **d**, delayed phase of CTHA. White arrowheads indicate staining of tumour. Two months after treatment: **e**, right hepatic angiography; **f**, plain CT image; **g**, early phase of dynamic CT; **h**, delayed phase of dynamic CT. Black arrow indicates remaining lipiodol.

Relatively good tumour control was recorded in one patient (case 3 in the level 1 group) who underwent RFA before response evaluation. Therefore, anti-tumour response was assessed in eight patients using computed tomography (CT) and tumour markers. Changes in the HCC diameter and levels of α-fetoprotein (AFP) and des-γ-carboxy prothrombin (DCP) following treatment are summarised in Table 3 and Figure 2. With a median follow-up period of 120, 87 and 83 days for level 1, 2 and 3 groups, respectively, case 9 in the level 3 group showed a partial response (PR) to therapy. Cases 1, 4, 6 and 8 showed stable disease response, while cases 2, 5 and 7 showed progressive disease response (Table 3). These changes were consistent with the changes recorded by the tumour markers (Figure 2). One patient (case 9) with multiple HCC in both lobes (Figure 3a–d), who showed resistance to previous treatment with DDP-H-TAC and epirubicin-TACE, evidenced a PR response following combination therapy of 65 mg/m^2^ of DDP-H-TAC and miriplatin-TOCE (Figure 3e–h). Significant reduction in HCC size in the right lobe was seen on right hepatic angiography (Figure 3a, e). A representative tumour in S6 showed no enhancement by CT during arterial portography (white arrow in Figure 3b), and significant enhancement in the early phase of CT hepatic arteriography was followed by ‘corona-like’ staining, which is a typical enhancement pattern seen in classical HCC (white arrowheads in Figure 3c, d) before treatment. Two months following treatment, the remaining lipiodol (black arrow in Figure 3f) and a marked decrease in tumour enhancement in the area were seen (Figure 3g, h).

**Table 3:** Anti-tumour effects: clinical efficacy

**Antitumor response**	**Level 1**	**Level 2**	**Level 3**
	**n, (Case number)**		
**CR**	0	0	0
**PR**	0	0	1, (Case 9)
**SD**	1, (Case 1)	2, (Case 4, 6)	1, (Case 8)
**PD**	1, (Case 2)	1 (Case 5)	1, (Case 7)
**Not evaluable**	1, (Case 3)	0	0
**DCR (%)**	50	66.7	66.7
**Period of follow up (day)**			
**Median**	120	87	83
**Range**	50-213	24-140	54-84

Modified Response Evaluation Criteria In Solid Tumors guidelines were followed to evaluate anti-tumour effects. CR, complete response; PR, partial response; SD, stable disease; PD, progressive disease; DCR, disease control rate.

## Discussion

Treatment for HCC was determined along with tumour stage and hepatic functional reserve, with only 30% of HCC cases being an indication of curative therapies such as surgical resection and RFA [2,6]. TACE and sorafenib have recently been reported to show a definite survival advantage in advanced cases [3,4,6-8,28]. Unfortunately, however, the application of TACE or sorafenib is strictly restricted by other factors, mainly hepatic functional reserve. TACE requires a Child–Pugh score of 5–9, grade A–B, for hepatic function as it involves arterial embolization and may not be completed in a patient with major arterioportal shunts or portal vein tumour thrombosis. Sorafenib is contraindicated in patients with the exceptions of Child–Pugh score of 5–6, grade A or with brain metastases [2]. In contrast, TOCE and TAC can be provided over a broad range of cases as these are performed without arterial embolization and their efficacy has been reported [5,13-15,26]. Among various chemotherapeutic agents such as epirubicin [15] and mitomycin C [5], which carry a 15%–20% response rate, platinum agents appear to be the most promising as CDDP-TAC achieved a response rate of 33.8% in a multicentre phase II study enrolling unresectable HCC cases [13]. To investigate the highly effective and less toxic combination of TOCE and TAC, this study focused on safety issues associated with the concomitant use of two platinum agents.

Miriplatin is a third-generation platinum agent with amphipathic properties that forms a stable suspension with lipiodol and gradually releases active derivatives *in situ*, which circumvents systematic release and toxicity [18]. Treatment in few HCC cases has shown cross-resistance with different generations of platinum agents [16,21,29]. In a rat model, miriplatin exhibited higher anti-tumour activity and lower hepatic toxicity than CDDP-lipiodol [16], and promising results have been reported in HCC patients [22-24]. On the other hand, clearance of platinum compounds following short-term intravenous infusion of cisplatin was reported as triphasic (distribution half-life, 13 min; elimination half-life, 43 min and terminal half-life, 5.4 days). The short distribution half-life suggests that TAC easily exceeds tissue distribution speed and saturates the target liver on the basis of concentration rather than the total amount of the drug administered. Accordingly, DDP-H is currently the most suitable form of platinum agent for TAC by providing the highest concentration available. The combination of DDP-H-TAC and miriplatin-TOCE supports the hypothesis that higher the free platinum concentration in the target liver, lesser the systemic spill over and more sustained delivery achieved by a less cross-resistant agent leads to marked tumour response and less toxicity (both systemic and hepatic), leading to improvement in survival rates.

## Conclusions

In conclusion, in this study, no DLT was recorded following the combined administration of DDP-H and miriplatin at a maximum dose of 65 mg/m^2^ and 120 mg/body, respectively. These are the maximum doses recommended for each monotherapy individually, indicating that the MTD of DDP-H and miriplatin in combination therapy is the maximum monotherapy dose. No evidence of systemic platinum release from miriplatin-TOCE was recorded, as expected. Reflecting a possible higher disease control rate and PR response, a phase II randomised prospective study is now ongoing to investigate the efficacy of this combined therapy in a larger cohort.

## Competing interests

The authors declare that they do not have a current financial arrangement or affiliation with any organisation that may have a direct interest in their work.

## Author’s contributions

KK wrote manuscript and performed research. TS designed a research and wrote manuscript. YT, MT, MI, HK, and SY performed research including the angiography. TY analysed data. MN and YA designed and analysed all data. All authors read and approved the final manuscript.

## Disclosure

The authors declare that they do not have a current financial arrangement or affiliation with any organisation that may have a direct interest in their work.

## Pre-publication history

The pre-publication history for this paper can be accessed here:

http://www.biomedcentral.com/1471-230X/12/127/prepub
